# Hemodiafiltration Treatment for Severe Valproic Acid Intoxication: Case Report and Updated Systematic Literature Review

**DOI:** 10.3389/fmed.2018.00224

**Published:** 2018-08-10

**Authors:** Tobias Tichelbäcker, Judith Herath, Björn Tampe, Peter Korsten

**Affiliations:** ^1^Department of Cardiology and Pneumology, University Medical Center, DZHK (German Centre for Cardiovascular Research), Göttingen, Germany; ^2^Department of Nephrology and Rheumatology, University Medical Center, Göttingen, Germany

**Keywords:** hemodialysis, intoxication, valproic acid, extracorporeal treatment, hemodiafiltration

## Abstract

**Background:** Valproic acid (VPA) has been approved for the treatment of seizure disorders. It is also commonly used in psychiatric disorders, such as schizophrenia spectrum disorders. With increasing administration, reports of intoxications are more frequently reported. The most common findings of VPA intoxication are central nervous system depression, respiratory depression, hypotension, metabolic acidosis, and elevated lactate, among others.

**Methods:** We describe a case report of VPA intoxication with hemodiafiltration (HDF) as extracorporeal treatment (ECTR) for removal of VPA. This treatment modality has only rarely been reported in the current literature. In addition, we performed an updated systematic literature review (SLR) of additional cases on the topic ranging from December 1st, 2014 to April 20th, 2018. We searched MEDLINE and Web of Science for relevant references.

**Results:** In the presented case, VPA intoxication occurred in a 46-year-old female patient after oral ingestion of 56 g of VPA. In addition to vasopressors and endotracheal intubation, we administered L-Carnitine (L-Car) and performed hemodiafiltration treatment. After intravenous therapy with L-Car and simultaneous HDF sessions, we observed full recovery without neurological sequelae. The SLR identified 8 additional articles reporting favorable outcomes with extracorporeal treatments in most cases.

**Conclusion:** HDF and other extracorporeal procedures are safe and effective therapeutic options in patients with VPA intoxication. The choice of ECTR modality mainly depends on local experience and the setting. In the present case, ingestion of 56 g was successfully treated with HDF. These findings are in line with several other case reports describing positive outcomes. Extracorporeal treatment, including HDF, should be considered early in the management of VPA intoxication. Supporting evidence is emerging, but it is of limited quality.

## Background

Valproic acid (VPA) is an approved treatment for seizure disorders and recommended by a recent Cochrane review (1). It is also increasingly used for psychiatric disorders, such as schizophrenia spectrum of disorders (2), and migraine prophylaxis (3). Recognized complications of VPA intoxication are central nervous system depression, acidosis, shock, acute hyperammonemia, cerebral edema, and it can lead to death (4). In addition to symptomatic measures for these complications, which include mechanical ventilation, administration of fluids or vasopressors, extracorporeal treatment (ECTR) strategies have been described in the treatment of valproate intoxication. The role of hemodialysis (HD) or hemodiafiltration (HDF) for VPA intoxication is controversial because about 90–95% is protein-bound at therapeutic levels and, as such, less amenable to extracorporeal removal procedures (5). We present the case of a severe intoxication with 56 g of VPA, in which we used HDF as a relatively novel extracorporeal treatment, and provide an updated systematic literature review of extracorporeal treatments in VPA intoxications in addition to a recently published systematic review of published studies and case reports (4).

## Case report

The patient, a 46-year old woman, was found unconsciously in her home. Upon arrival of the emergency medical service personnel, a Glasgow Coma Scale of 5 was present, the patient was intubated immediately and transferred to our intensive care unit. We assumed ingestion of 56 g of valproate based on the emptied medication boxes found by emergency medical services. Laboratory testing confirmed very high levels of valproate acid at >10389.5 μmol/l (normal range (NR) of therapeutic levels: 346.5–693.0 μmol/l). Additionally, a blood alcohol concentration of 1.18%0 was detected. The concentration of ammonia was slightly elevated (197 μg/ml, NR: 31–123 g/dl). Apart from slightly elevated uric acid (7.8 mg/dl, NR: 2.6–6.0 mg/dl), which was deemed to be clinically insignificant, all other laboratory values were within the normal range.

We initiated intravenous therapy with L-Carnitine (L-Car) with a loading dose of 100 mg/kg, followed by 50 mg/kg eight and 16 h later. Simultaneously, we performed extracorporeal removal with HDF. Elimination was performed using a high-flux dialyzer (FX60 CorDiax, Fresenius Medical Care) with two HDF sessions of 12 h duration per treatment, interrupted by a 10-h break. Valproate elimination was monitored by measurements of drug concentrations every 6 h. After two treatments, a serum concentration of 255.4 μmol/l was obtained and serum ammonia levels normalized.

**Figure 1:**
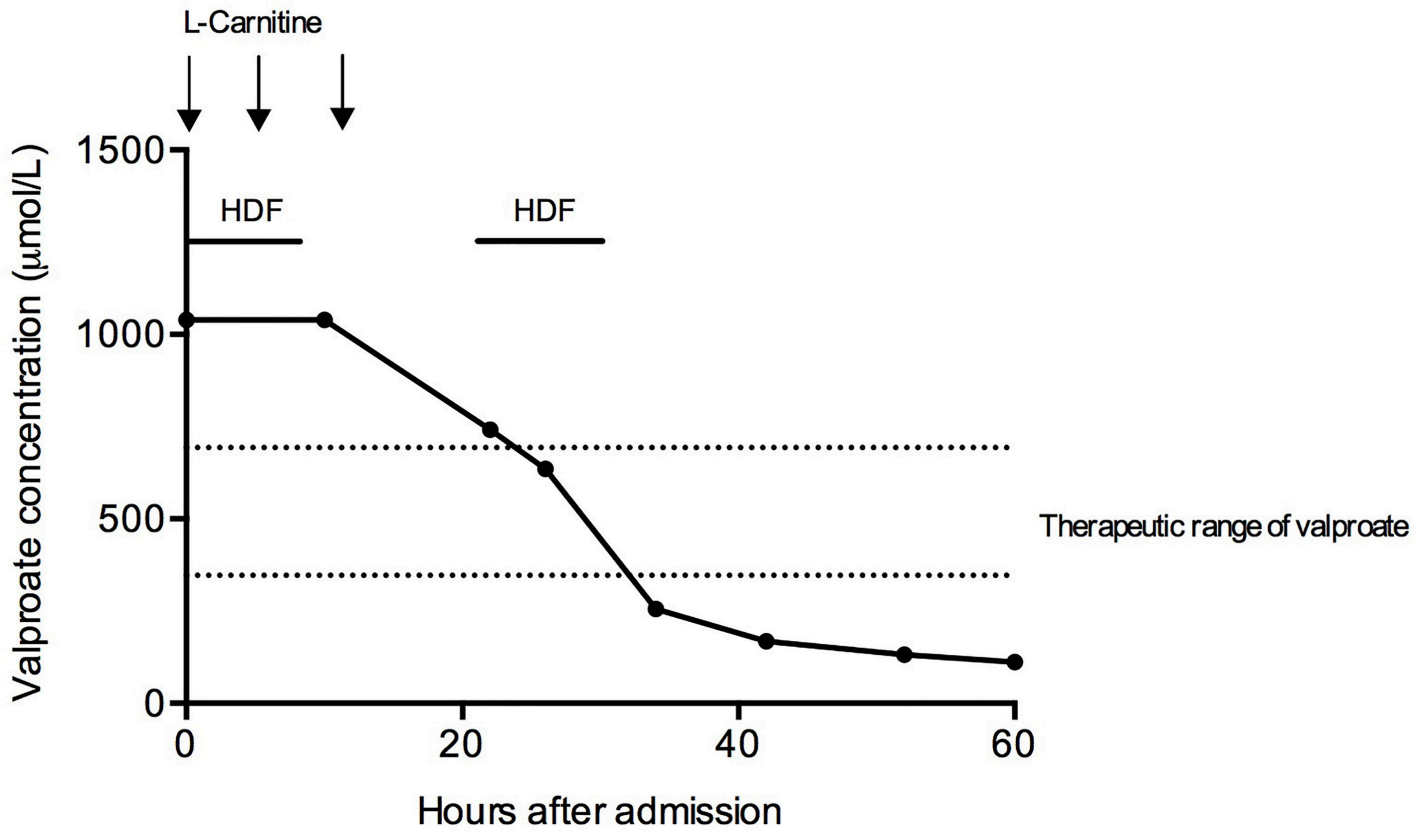
Treatment course of the patient. Hemodiafiltration and L-carnitine infusions were initiated on admission. Two hemodiafiltration sessions of 12-h duration were initiated. HDF, hemodiafiltration.

Therefore, we stopped HDF treatment and further measurements 12 and 24 h later confirmed decreasing drug concentrations (Figure 1). The patient's mental status improved and she was extubated 12 h after admission to the ICU. She finally was transferred to a psychiatric facility due to continued suicidal ideation but without neurological sequelae.

## Updated systematic review of the literature

**Figure 2:**
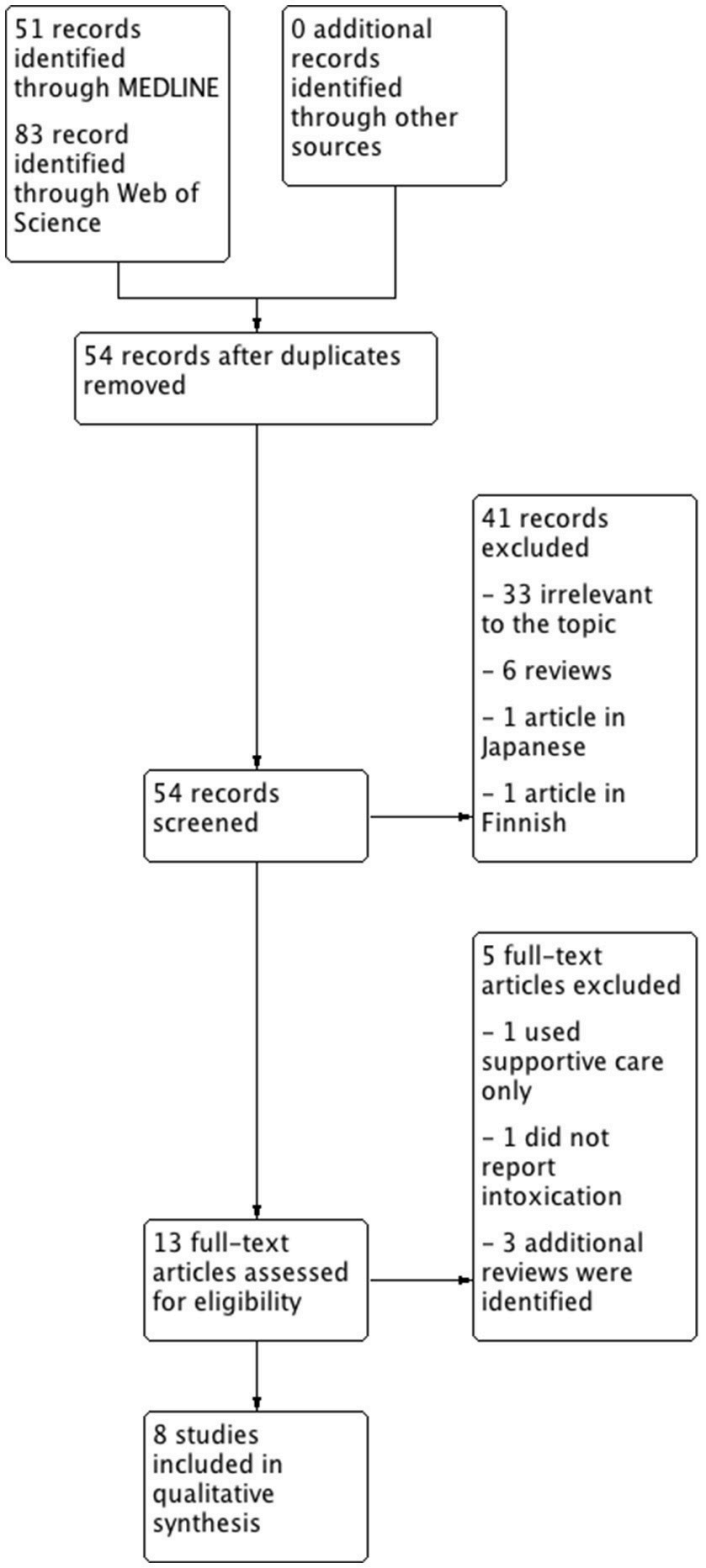
Flowchart of the systematic literature review.

We searched MEDLINE and Web of Science with the same search strategy (see appendix for detailed information) as Ghonnoum and coworkers in a recently published systematic review of the literature of ECTR in VPA intoxications (4) with dates ranging from December 1st, 2014 until April 20th, 2018. This date range was chosen, because papers published until November 2014 were covered in the literature search of Ghannoum et al. Articles in English, German, or Spanish were deemed eligible for further review since the authors are able to read and understand these languages. Articles published in peer-reviewed journals were considered relevant. Review articles and articles written in other languages than those mentioned were excluded. We identified 134 papers with this search strategy. After exclusion of papers according to the pre-specified criteria, eight articles were included in the literature review (Figure 2). A summary of the included cases, the treatment strategies, and outcomes are presented in Table 1.

**Table 1:** Overview of published cases of VPA intoxication treated with extracorporeal removal procedures.

**First Author**	**Year**	**N of patients**	**Ingested dose of VPA**	**Treatment**	**Outcome**
(6)	2018	1	60 g	AC, L-Car, CRRT, HD	Full recovery
**Present case**	**2017**	**1**	**56 g**	**HDF, L-Car**	**Full recovery**
(7)	2017	1	20 g	AC, L-Car, FPSA-CVVH	Full recovery
(8)	2017	5	18 – 56 g	AC, L-Car, L-Arg, HD	Full recovery
(9)	2015	316	average 6,5 g	AC, HD in 3 patients	Full recovery n = 302 Death n = 2
(10)	2015	1	Unknown	HP	Full recovery
(11)	2014	1	14,5 g (+14,6 g carbamazepine)	AC, HP followed by CVVH	Full recovery
(12)	2014	1	Unknown	PD	Full recovery
(13)	2014	1	60 g	AC, HD	Full recovery

*AC, activated charcoal; CRRT, continuous renal replacement therapy; CVVH, continous venovenous hemofiltration; FPSA-CVVH, fractionated plasma separation and adsorption with continuous venovenous hemofiltration; HD, hemodialysis; HDF, hemodiafiltration; HP, hemoperfusion; L-Arg, L-Arginine, L-Car, L-carnitine, PD-peritoneal dialysis*.

### Description of the published cases

The presented cases (6–13) reported intoxication doses ranging from 6.5 to 60 g (Table 1). Six cases were single case reports; one article described a case series of five patients, the most extensive analysis reviewed 316 patients with VPA intoxication. In almost all of the cases, a favorable outcome with full recovery was reported. Shadnia et al. described two deaths due to VPA intoxication in their retrospective study of 316 patients in Iran (9). Most patients were intubated due to severe CNS depression. ECTRs varied between the cases, and hemodialysis (HD), hemodiafiltration (HDF), hemoperfusion (HP), continuous renal replacement therapy (CRRT), and liver support therapy were all used. Kumar et al. demonstrated the feasibility of peritoneal dialysis in an acute setting (12).

## Discussion

In the case we present here, the assumed amount of ingested VPA was 56 g, which is, compared with other cases, at the upper end of the dose range in the reported cases. Mean ingested dose ranged from 4 to 160 g in the analysis by Ghannoum et al. (4), in our additional cases doses ranged from 6.5 to 60 g (Table 1). Also, our patient required intubation due to severe CNS depression, which is almost universal in severe intoxications (96.3%), according to data analyzed by the EXTRIP working group (4). The laboratory findings we encountered in our patient (mild hyperammonemia) have also been described in the literature. Additional clinical features and complications reported include (in descending frequency): Respiratory depression (65.9%), hypotension (39.0%), metabolic acidosis (28.0%), elevated lactate (23.2%), thrombocytopenia (14.6%), seizures (11.0%), cerebral edema (7.3%), and hypernatremia (4.9%) (4).

Management of mild VPA intoxications is usually supportive. General measures of gastric decontamination with activated charcoal may be considered when ingestion is observed or admission to healthcare facilities occurs shortly after ingestion. Intravenous fluids or vasopressors may be necessary in hypotensive patients (4). Additional measures often recommended include L-Car, which is thought to act beneficially on mitochondrial dysfunction, but the overall evidence for L-Car is limited (14, 15). However, it appears that L-Car has hardly any adverse effects with the exception of hyophosphatemia, which has rarely been reported (16).

We used HDF as treatment modality relatively early in the treatment course. This needs some explanation and considerations: For a drug to be removed by ECTRs, it should ideally be of low molecular weight, highly water-soluble and not bound to plasma proteins (17). Keeping these pharmacological properties in mind, VPA is not easily removed by ECTRs because it is highly protein-bound at therapeutic levels (4, 17). At supra-therapeutic levels, however, VPA is less protein-bound and therefore circulating freely in plasma (4). The analysis by the EXRIP resulted in recommendations and suggestions for the use of ECTRs (4): (1) ECTR is recommended if VPA concentrations exceed 1,300 mg/l OR if cerebral edema or shock attributable to VPA toxicity are present. (2) ECTRs suggested in patients with VPA concentrations >900 mg/l, coma or respiratory depression requiring mechanical ventilation, hyperammonemia, or pH < 7.10. The modality of ECTR itself seems to be, according to the reported literature, not a very important factor in the treatment of VPA intoxicated patients since many different modalities have been described with success. Nevertheless, we used HDF as a relatively novel treatment modality. This was based on our personal preference/experience.

## Concluding remarks

In conclusion, we report the successful use of high-flux hemodiafiltration and L-carnitine treatment in a patient with severe acute VPA intoxication. Evidence for extracorporeal removal strategies in VPA intoxications is emerging as evidenced by a recent systematic review and by our updated systemic review on the topic. The type of ECTR should be chosen according to personal preference, experience and local circumstances. Ideally, ECTRs in VPA intoxications should be investigated in a controlled clinical trial setting, but this is difficult to plan/perform since the overall occurrence of VPA intoxications per center will be limited to few cases. ECTRs appear to be safe and deaths by VPA intoxications are only rarely reported with adequate treatment.

## Ethics statement

Informed consent was obtained from the patient.

## Author contributions

TT and JH treated the patient, co-wrote the manuscript and analyzed data. TT and JH contributed equally as first authors. BT analyzed data, performed the literature search and the systematic review, drafted the figures, and co-wrote the manuscript. PK conceived the study, treated the patient, performed the literature search and the systematic review, drafted the figures and co-wrote the manuscript. BT and PK contributed equally as senior authors.

### Conflict of interest statement

The authors declare that the research was conducted in the absence of any commercial or financial relationships that could be construed as a potential conflict of interest.

The reviewer FG and handling Editor declared their shared affiliation.
