# Supplementary material for: Hemodiafiltration Treatment for Severe Valproic Acid Intoxication: Case Report and Updated Systematic Literature Review
Source: Front Med (Lausanne). 2018 Aug 10;5:224. doi: 10.3389/fmed.2018.00224 (PMC6095960; doi:10.3389/fmed.2018.00224)
Supplement: Supplementary file 1 [file Table_1.DOCX]

**Appendix:**

Search strategy for MEDLINE and Web of Science:

[(valpro*) AND (dialysis OR hemodialysis OR haemodialysis OR hemoperfusion OR haemoperfusion OR plasmapheresis OR plasma exchange OR exchange transfusion OR hemofiltration OR haemofiltration OR hemodiafiltration OR haemodiafiltration OR extracorporeal therapy OR CRRT)]

The search strategy was the same, which Ghannoum and coworkers used for their systematic review [3]. Our literature search was performed ranging from December 1^st^ 2014 until April 20^th^ 2018.
